# Prediction-oriented prognostic biomarker discovery with survival machine learning methods

**DOI:** 10.1093/nargab/lqad055

**Published:** 2023-06-16

**Authors:** Sijie Yao, Biwei Cao, Tingyi Li, Denise Kalos, Yading Yuan, Xuefeng Wang

**Affiliations:** Department of Biostatistics and Bioinformatics, H. Lee Moffitt Cancer Center & Research Institute, Tampa, FL 33612, USA; Department of Biostatistics and Bioinformatics, H. Lee Moffitt Cancer Center & Research Institute, Tampa, FL 33612, USA; Department of Biostatistics and Bioinformatics, H. Lee Moffitt Cancer Center & Research Institute, Tampa, FL 33612, USA; Department of Biostatistics and Bioinformatics, H. Lee Moffitt Cancer Center & Research Institute, Tampa, FL 33612, USA; Department of Radiation Oncology, Icahn School of Medicine at Mount Sinai, New York City, NY 10029, USA; Department of Biostatistics and Bioinformatics, H. Lee Moffitt Cancer Center & Research Institute, Tampa, FL 33612, USA

## Abstract

Identifying novel and reliable prognostic biomarkers for predicting patient survival outcomes is essential for deciding personalized treatment strategies for diseases such as cancer. Numerous feature selection techniques have been proposed to address the high-dimensional problem in constructing prediction models. Not only does feature selection lower the data dimension, but it also improves the prediction accuracy of the resulted models by mitigating overfitting. The performances of these feature selection methods when applied to survival models, on the other hand, deserve further investigation. In this paper, we construct and compare a series of prediction-oriented biomarker selection frameworks by leveraging recent machine learning algorithms, including random survival forests, extreme gradient boosting, light gradient boosting and deep learning-based survival models. Additionally, we adapt the recently proposed prediction-oriented marker selection (PROMISE) to a survival model (PROMISE-Cox) as a benchmark approach. Our simulation studies indicate that boosting-based approaches tend to provide superior accuracy with better true positive rate and false positive rate in more complicated scenarios. For demonstration purpose, we applied the proposed biomarker selection strategies to identify prognostic biomarkers in different modalities of head and neck cancer data.

## INTRODUCTION

The fast advancement of genomic sequencing and other high-throughput molecular profiling technologies has made it possible to characterize tens of thousands of genes and biomarkers at the same time. Due to the high dimensionality and complex data dependence structure, deploying genome-wide biomarker screening has posed both statistical and computational challenges. In statistics and machine learning modeling, the search for biomarkers can be viewed as feature selection or variable selection in a regression model. The purpose of this article is to discuss the analysis of prognostic biomarkers with the main goal of identifying markers that are associated with and can be predictable for patient survival outcomes. Multivariable survival analysis is complicated by the problem of censoring, which occurs when the patient survival time after the date of diagnosis is only known in part. Another critical but frequently overlooked fact is that all survival models are built based on certain distributional assumptions about survival time.

The Cox proportional hazards regression model (1) has been one of the most widely used tools for survival analysis in biomedical research, but its traditional implementation is not suitable for analyzing modern genomic data, where the number of candidate biomarkers is much greater than the sample size. Various feature selection techniques have been developed to address the high-dimensional problem. One of the most commonly used techniques is to apply penalization constraints to the original likelihood or objective functions and thereby generate sparse solutions, such as the lasso (2) and elastic net (3) penalty terms. By incorporating lasso or elastic net with a combined cross-validation (CV) and stability selection (SS) procedure for parameter turning, Kim *et al.* (4) proposed a method called prediction-oriented marker selection (PROMISE). Another popular solution for solving high-dimensional problems in machine learning is to use tree-based boosting algorithms such as extreme gradient boosting (XGB) (5) and light gradient boosting (LGB) (6), which are well known for their high computational efficiency and exceptional predictive performance when accommodating nonlinear effects. Both XGB and LGB have been recently extended and evaluated in the context of a survival model (7). Based on the deep learning architecture, the study by Katzman *et al.* (8) proposed a Cox deep neural network (DeepSurv) method, where the Cox likelihood loss was implemented in a multilayer feedforward network. While individual benchmark studies have reported promising results, there are few studies directly comparing the performances of these advanced survival models in biomarker selection and survival outcome prediction.

**Figure 1.**
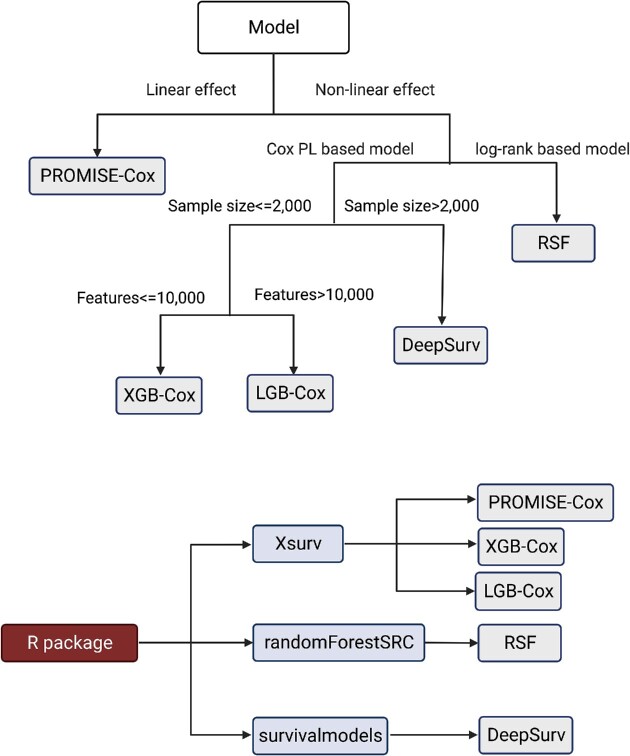
Workflow of selecting the most suitable prediction-oriented machine learning methods and corresponding R packages used for different machine learning methods.

The purpose of this article is to conduct a systematical evaluation of the performances of most widely used survival machine learning methods under a variety of scenarios, with a particular emphasis on predictive biomarker prioritization. Our work is motivated by an increasing demand for guidance and standards regarding the deployment of prognostic biomarker discovery analysis pipelines in cancer research, where survival outcomes are one of the most critical clinical outcomes or endpoints. Five representative machine learning approaches were chosen as a result. In general, the primary goal of most machine learning methods is to provide high prediction accuracy. However, the feature selection results may not be clearly clarified, especially in nonlinear machine learning models. Many machine learning software packages provide feature importance as an option for feature selection, but they do not give a threshold to determine the final decision. Although the feature selection results can be directly determined by the coefficients in linear models, the results can still become unreliable due to excessive false positives. To reduce the number of false positives and keep the high prediction accuracy, the study by Kim *et al.* (4) proposed PROMISE that combines CV and SS together in generalized linear models. To deal with the survival data, we extend PROMISE from the generalized linear model to the Cox model **(**PROMISE-Cox). Furthermore, in order to identify the significant features in nonlinear machine learning models, we propose a prediction-oriented feature selection algorithm that combines CV and top-*k* selection. The description of the proposed algorithm with pseudo-code can be found in Supplementary Table S2. We use ‘XGB-Cox-select’, ‘LGB-Cox-select’, ‘RSF-select’ and ‘DeepSurv-select’ to denote the machine learning-based selection models. For the sake of convenience, we use only the machine learning models’ names to represent the machine learning-based variable selection framework throughout this paper. We implement the feature selection procedure in ‘Xsurv’ with function ‘x_select’. Different machine learning methods can be used in ‘x_select’ by choosing the corresponding option in ‘method’ argument. In this study, we focus on five different machine learning methods, which can be mainly divided into two categories: linear model and nonlinear model. A summarization for the different machine learning methods is presented in Figure 1 based on simulations and experience in a previous work (7). We evaluate different machine learning approaches in four simulation scenarios: (i) linear model; (ii) quadratic model; (iii) nonlinear model without interactions and (iv) nonlinear model with interactions. Finally, we demonstrate the utility of these methods by applying them to different modalities of head and neck cancer data, followed by discussion.

## MATERIALS AND METHODS

### Cox model

In this paper, we focus on the Cox proportional hazards (1) based model. In survival analysis, we consider the time-to-event (e.g. time to death) data $( {{t}_i,{{\boldsymbol{x}}}_i,\ {\delta }_i} ),$ where ${t}_i$ is the observed event time, ${{\boldsymbol{x}}}_{\boldsymbol{i}}$ is the covariate (feature) vector, ${\delta }_i$ is the censoring indicator and *i* indices samples from 1 to *N*. The Cox model defines the hazard function as follows:


$$\begin{equation*}\lambda \left( {t{\rm{|}}{x}_i} \right) = {\lambda }_0\left( t \right)\exp \left\{ {H\left( {{{\boldsymbol{x}}}_{\boldsymbol{i}}} \right)} \right\},\end{equation*}$$

where ${\lambda }_0( t )$ is the baseline hazard and $H( \cdot )$ is a risk score function determined by the covariates. In the standard Cox model, the risk score is expressed as a linear function, i.e. *H*$( {{x}_i} ) = {\boldsymbol{x}}_i^T{\boldsymbol{\beta }}$. To estimate the regression coefficient ${\boldsymbol{\beta }}$, the loss function is defined by the negative log partial likelihood (PL) plus a penalty:


$$\begin{equation*}{\boldsymbol{\hat{\beta }}} = \mathop {{\rm{arg\ min}}}\limits_{\boldsymbol{\beta }} \left[ { - {\rm{log\,PL}} + {P}_\lambda \left( {\boldsymbol{\beta }} \right)} \right],\end{equation*}$$

where ${\rm PL} = \mathop \prod \nolimits_i {\big[ {{{{\rm{exp}}( {H( {{x}_i} )} )}}/{{\mathop \sum \nolimits_{k\epsilon R( {{t}_i} )} {\rm{exp}}( {H( {{x}_k} )} )}} } \big ]}^{{\delta }_i}$ and ${P}_\lambda ( {\boldsymbol{\beta }} )$ is the penalty function, such as the L1 penalty or an elastic net penalty. Here, $R( {{t}_i} )$ is the set of the observations at risk at time ${t}_i$.

### Prediction-oriented selection method

In general, the primary goal of most machine learning methods is to provide high prediction accuracy. However, the feature selection results may not be clearly clarified, especially in nonlinear machine learning models. Many machine learning software packages provide feature importance as an option for feature selection, but they do not give a threshold to determine the final decision. Although the feature selection results can be directly determined by the coefficients in linear models, the results can still become unreliable due to excessive false positives. To reduce the number of false positives and keep the high prediction accuracy, the study by Kim *et al.* (4) proposed PROMISE that combines CV and SS together in generalized linear models. To deal with the survival data, we extend PROMISE from the generalized linear model to the Cox model (PROMISE-Cox). Furthermore, in order to identify the significant features in nonlinear machine learning models, we propose a prediction-oriented feature selection algorithm that combines CV and top-*k* selection. The description of the proposed algorithm with pseudo-code can be found in Supplementary Table S1. We implement the feature selection procedure in ‘Xsurv’ (7) with function ‘x_select’. Different machine learning methods can be used in ‘x_select’ by choosing the corresponding option in ‘method’ argument. In this study, we focus on five different machine learning methods, which can be mainly divided into two categories: linear model and nonlinear model. A summarization for the different machine learning methods is presented in Figure 1. To convey the main idea of different machine learning methods, we give a brief description of them in the following.

The model for PROMISE-Cox is trained by a linear objective function with a lasso (L1) (2) or elastic net penalty (3). The lasso penalty can be written as


$$\begin{equation*}{P}_\lambda \left( {\boldsymbol{\beta }} \right) = \lambda \mathop \sum \limits_{j\ = \ 1}^p \left| {{\beta }_j} \right|\end{equation*}$$

and the elastic net penalty is a linear combination of L1 and L2 (ridge penalty) (9) penalties:


$$\begin{equation*}{P}_{\alpha ,\lambda }\left( {\boldsymbol{\beta }} \right) = \lambda \mathop \sum \limits_{j\ = \ 1}^p \left\{ {\alpha \left| {{\beta }_j} \right| + \frac{1}{2}\left( {1 - \alpha } \right){{\left| {{\beta }_j} \right|}}^2} \right\}.\end{equation*}$$

The significant features or markers are identified with nonzero regression coefficients. The pseudo-code for PROMISE-Cox can be found in Supplementary Table S1. We implement the PROMISE-Cox model in the R package ‘Xsurv’ in function ‘x_proms’.

Decision tree is a widely used machine learning technique for nonlinear models. In this paper, we introduce two representative decision tree models: gradient boosting decision tree and random forests. XGB and LGB are two modern gradient boosting models and have been implemented for survival data recently (7). Both XGB-Cox and LGB-Cox use the negative partial likelihood as the loss function. In gradient boosting, the loss function is iteratively optimized by finding a weak learner that is nearest to the negative gradients. We denote the gradient and second derivative of the Cox PL loss by ${g}_i$ and ${s}_i$, respectively. The optimal basis function at the *m*th step ${\eta }^{( m )}$ can be written as


$$\begin{equation*}{\eta }^{\left( m \right)} = \mathop {{\rm{arg\ min}}}\limits_{\eta \in H} \mathop \sum \limits_{i = 1}^N \left( {\frac{1}{2}s_i^{\left( m \right)}{{\left[ { - \frac{{g_i^{\left( m \right)}}}{{s_i^{\left( m \right)}}} - {\eta }^{\left( m \right)}\left( {{{\boldsymbol{x}}}_{\boldsymbol{i}}} \right)} \right]}}^2} \right).\end{equation*}$$

Hence, the risk score function can be updated by ${H}^{( m )} = {H}^{( {m - 1} )} + \epsilon {\eta }^{( m )}$, where $\epsilon$ is the learning rate. In practice, $\epsilon$ is often set around 0.001 and we provide the frequently used searching values for $\epsilon$ in Table 3. In particular, high computational efficiency is achieved with boosting tree frameworks (7). Furthermore, to avoid overfitting, XGB/LGB incorporate the regularization terms into the loss function. The main difference between XGB and LGB is that they use different tree construction strategies. XGB uses the level-wise strategy, while LGB employs a leaf-wise growth strategy for tree construction and the gradient-based one-side sampling to force a split. Although XGB with the Cox loss is implemented in the ‘xgboost’ package with the objective option ‘survival:cox’, it is limited by the evaluation metric that only contains the PL loss function. In addition, there is no particular function for ‘LightGBM’ package on survival outcomes. We use the functions from ‘Xsurv’ package, in which both XGB and LGB are implemented on survival outcomes with different evaluation metrics [the PL loss and concordance index (*C*-index)]. Random forest (10) is another type of decision tree that predicts the results with the entire forest. Random survival forests (RSF) (11) is an extension of random forests in time-to-event survival data. In an RSF, each tree is constructed independently with a randomly drawn bootstrap sample. Different from PROMISE, XGB and LGB, RSF modeling does not depend on the Cox loss function. We use the function ‘rfsrc’ in the R package ‘randomForestSRC’ in our study. The splitting rule used by the package is the log-rank test statistic (12–14). The maximization of the log-rank split-statistic value ensures the largest survival difference between left and right daughter nodes.

Over the last decade, deep learning methods that are based on artificial neural networks draw a growing attention in machine learning field for the abilities in analyzing the structures of high-dimensional data in various areas such as image recognition (15–18). To apply the deep learning methods in survival data, a Cox model-based neural network called DeepSurv was proposed by Katzman *et al.* (8). DeepSurv is a feedforward neural network that is trained by the Cox PL with regularization terms as the objective function. The risk score function $H( \cdot )$ is then the function of network weights. The hidden layers of DeepSurv are constructed with a fully connected layer of nodes. To prevent the overfitting issue, each layer is followed by a dropout layer (19). The final output of DeepSurv is a single node that estimates the risk score function $H( \cdot )$. In addition, DeepSurv proposed a treatment recommender system that can be used to predict $H( \cdot )$ by given treatment groups in a clinical study. To improve the network performance, several deep learning techniques have been employed in DeepSurv, such as scaled exponential linear units (20), adaptive moment estimation (21) and learning rate scheduling (22). We use the function deepsurv from the R package ‘survivalmodels’ to fit DeepSurv networks.

In general, different machine learning methods have their own strengths and limitations. The knowledge of the algorithms is important in order to decide the best suited method based on the application. We summarize some main advantages and disadvantages of the machine learning methods we introduced in this section in Table 1.

**Table 1.** An overview of advantages and disadvantages of different machine learning methods in survival analysis

Algorithm name	Advantages	Disadvantages
PROMISE-Cox	• Good performance in small samples • Good performance for the linear case	• Missing data are not allowed • Poor performance for the nonlinear case
XGB/LGB	• High computational efficiency • Allows missing data • Good performance for the nonlinear case • Good in high-dimensional data	• Sensitive to outliers • Easy to overfit
RSF	• Allows missing data • Little effect with model misspecification	• Interpretability of ensemble • Requires much computational power
DeepSurv	• Good performance for the nonlinear case • Provides solutions with treatment effect in clinical studies	• Requires a large sample size for good performance • Missing data are not allowed

### Hyperparameter tuning

One of the most important things for machine learning methods is how to deal with the hyperparameter tuning. The optimal parameters are determined by the CV procedure included in feature selection algorithms for PROMISE-Cox and nonlinear machine learning models. Two parameter searching schemes are applied: grid search and random search. In particular, some machine learning models are not very sensitive to some specific hyperparameters, such as L1/L2 regularization terms in XGB-Cox or LGB-Cox. Therefore, we can improve the computational efficiency by reducing the size of searching space. We offer the recommended setting of parameter values for different hyperparameters of the machine learning methods in Table 2.

**Table 2.** Recommended ranges of key hyperparameters

Algorithm name	Parameters	Value
PROMISE-Cox	Lasso parameter: $\lambda$	0.01–0.1
	Elastic net parameter: $\alpha$	0.05–0.5
	SS threshold	0.6–0.8
	Bootstrap sample size	100
XGB and LGB	Learning rate: ‘eta’	0.001, 0.005, 0.01, 0.05
	Subsample fraction: ‘frac’	0.5
	L1 regularization term: ‘lambda’	0.01–0.1
	L2 regularization term: ‘alpha’	0.01–0.1
	Number of trees: ‘nrounds’	100, 500, 1000
RSF	Number of trees: ‘n_estimate’	100, 500, 1000
	Minimum weighted fraction: ‘min_weight_fraction’	0.25, 0.5
DeepSurv	Validation sample fraction	0.2
	Number of nodes	2, 4, 8, 16, 32
	Number of layers	2, 4
	Dropout probability	0.1–0.6
	Epochs	20–50

### Evaluation metrics

Because of the censoring of survival data, it is difficult to evaluate survival models (23). To assess the survival model performance, there are some evaluation metrics proposed for survival analysis. The *C*-index (24) is the most widely used evaluation metric for survival analysis. Specifically, the *C*-index measures the prediction accuracy from concordant pairs. The definition of *C*-index is given by


$$\begin{equation*}C = \frac{1}{{\left| \mathcal{P} \right|}}\mathop \sum \limits_{\left( {i,j} \right) \in \mathcal{P}} 1\{ H\left( {{{\boldsymbol{x}}}_{\boldsymbol{i}}} \right) < H\left( {{{\boldsymbol{x}}}_{\boldsymbol{j}}} \right)\}, \end{equation*}$$

where $\mathcal{P}$ is the set of orderable pairs and ${t}_i < {t}_j$. *C*-index takes the value from 0 to 1, and a higher *C*-index indicates a better prediction performance.

Another commonly used evaluation metric is integrated Brier score (IBS) (25). Brier score (BS) can be calculated as


$$\begin{equation*}{\rm BS}\left( t \right) = \frac{1}{N}\mathop \sum \limits_{i\ = \ 1}^N \left[ {\frac{{1\{ {t}_i < t,{\delta }_i = 1\} \hat{S}{{\left( {t,{x}_i} \right)}}^2}}{{\hat{G}\left( {{t}_i} \right)}} + \frac{{1\{ {t}_i >t\} {{\left( {1 - \hat{S}\left( {t,{x}_i} \right)} \right)}}^2}}{{\hat{G}\left( t \right)}}} \right],\end{equation*}$$

where $\hat{S}( \cdot )$ is the survival function predicted by the model and $\hat{G}( \cdot )$ is the survival function corresponding to censoring, i.e. $\hat{G}( t ) = P({\rm Cen} >t)$, where Cen is the censoring time. IBS is then defined as the integral form of BS:


$$\begin{equation*}{\rm IBS} = \frac{1}{{{t}_{{\rm max}}}}\mathop \smallint \limits_0^{{t}_{{\rm max}}} {\rm BS}( t ){\rm d}t .\end{equation*}$$

In this study, to demonstrate the idea of the feature selection framework, we present the results using *C*-index as the evaluation metric.

### Simulations

We generated four simulation scenarios with varied model complexity. We begin with the Cox model with a simple linear link function in scenario 1 (linear model). More specifically, let covariates $X = {X}_1, \ldots ,{X}_p$ be i.i.d. standard normal distributed random variables, then the failure time $T$ follows an exponential distribution with mean at ${\rm{exp}}( {{X}_1 + {X}_2 + \cdots +{X}_q} ),$ where *p* is the total number of features and *q* is the number of true signals. The censoring time *C* follows an exponential distribution with a mean of $q$. Scenario 2 (quadratic model) is based on a quadratic function; i.e. the failure time *T* follows an exponential distribution with mean ${\rm{exp}}({1}/{2})( {X_1^2 + X_2^2 + \cdots + X_q^2} )$. In scenario 3 (nonlinear model without interactions), the failure time *T* follows an exponential distribution with a mean of


$$\begin{eqnarray*}&&{\rm{exp}}\{ 2\left[ {{\rm{\Phi }}\left( {[{X}_1 >0.5} \right] + X_2^2 - 1} \right)+{\rm{\ \Phi }}\left( {0.5{X}_3 + X_4^2 - 1} \right) \nonumber \\&& \ + \ {\rm{\Phi }}\left( {0.5{X}_5 + X_6^2 - 1} \right) + {\rm{ \Phi }}\left( {\sin {X}_7 + X_8^2 - 1{\rm{\ }}} \right) \nonumber \\ && \ + \ {\rm{\Phi }}\left( {\cos {X}_9 + X_{10}^2 - 1} \right)] + {X}_{11} + \cdots +{X}_q\} , \end{eqnarray*}$$

where ${\rm{\Phi }}$ is the standard normal cumulative distribution function. Here, $10 \le q \ll p$. The censoring time has a 1/3 chance to be 0.02 and a 2/3 chance to be uniform (0, 0.02), and the censoring rate in this case is ∼30%. In scenario 4 (nonlinear model with interactions), the failure time *T* follows a Weibull distribution with a mean of


$$\begin{eqnarray*}&&{\rm{exp}}\{ 4\left[ {{\rm{\Phi }}\left( {[{X}_1 >0.4} \right] \cdot X_2^2 - 1} + {\rm{\Phi }}\left( {0.6{X}_3 + X_4^2 - 1} \right)\right)\nonumber \\ && \ +\ {\rm{\Phi }}\left( {0.5{X}_5 \cdot {\rm{sin}}\,X_6^2 - 1} \right) + {\rm{\ \Phi }}\left( {\cos {X}_7 + X_8^2 - 1{\rm{\ }}} \right) \nonumber \\ &&\ +\ {\rm{\Phi }}\left( {\sin {X}_9 \cdot X_{10}^2 - 1} \right)] + {X}_{11} + \cdots +{X}_q\} .\end{eqnarray*}$$

Similar to the simulations described above, the failure time *T* for risk group classification simulation follows a Weibull distribution, with a shape parameter of 2 and the scale parameter


$$\begin{eqnarray*} && \upsilon \ = \ 3{\rm{\{ \Phi }}\left( {[{X}_1 >10} \right] + X_2^{} - 1{\rm{)}} + {\rm{\ \Phi }}(0.5{X}_3 + [X_4^{} > 5] - 1)\nonumber\\ && \ \ \ +\ {\rm{\Phi }}\left( {[{X}_5 > 10} \right] + [X_6^{} > 15]) + {\rm{\ \Phi }}\left( {\left[ {{X}_7 > 20} \right] + X_8^2 - 1{\rm{\ }}} \right)\nonumber\\ && \ \ \ +\ {\rm{\Phi }}([{X}_9 > 20] + X_{10}^2 - 1)\} .\end{eqnarray*}$$

The risk level is determined by the value of $\upsilon$ with a larger value of $\upsilon$ corresponding to a worse survival (Supplementary Figure S2). Based on the values of $\upsilon$, patients can be classified as high-risk group or low-risk group. The training data consisted of 1000 random samples, whereas the test data consisted of 200 samples.

## RESULTS

In this section, we assess the performances of discussed machine learning approaches for survival analysis using a suite of simulation settings and demonstrate the applications of these methods using a head and neck cancer dataset. In addition to prognostic predication, evaluating the effectiveness of biomarker identification in either targeted or genome-wide screening is an important component of our attention.

### Prognostic feature selection

**Figure 2.**
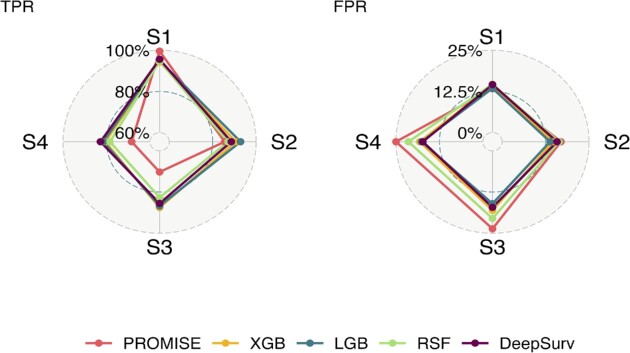
Biomarker selection results of different machine learning methods in the low-dimensional case: TPR (left panel) and 1 − FPR (right panel) with different scenarios: linear model (S1: scenario 1), quadratic model (S2: scenario 2), nonlinear model without interactions (S3: scenario 3) and nonlinear model with interactions (S4: scenario 4).

The results of feature selection when we set *N* = 1000, *p* = 100 and *q* = 10 are shown in Figure 2. We use the true positive rate (TPR) and the false positive rate (FPR) to characterize the performance of feature selection:


$$\begin{equation*}{\rm{TPR = }}\frac{{\# {\rm{\ true\ signals\ selected}}}}{{\# \ {\rm{total\ true\ signals}}}},\qquad {\rm{FPR = }}\frac{{\# {\rm{\ false\ signals\ selected}}}}{{\ \# \ {\rm{total\ false\ signals}}}}.\end{equation*}$$

**Figure 3.**
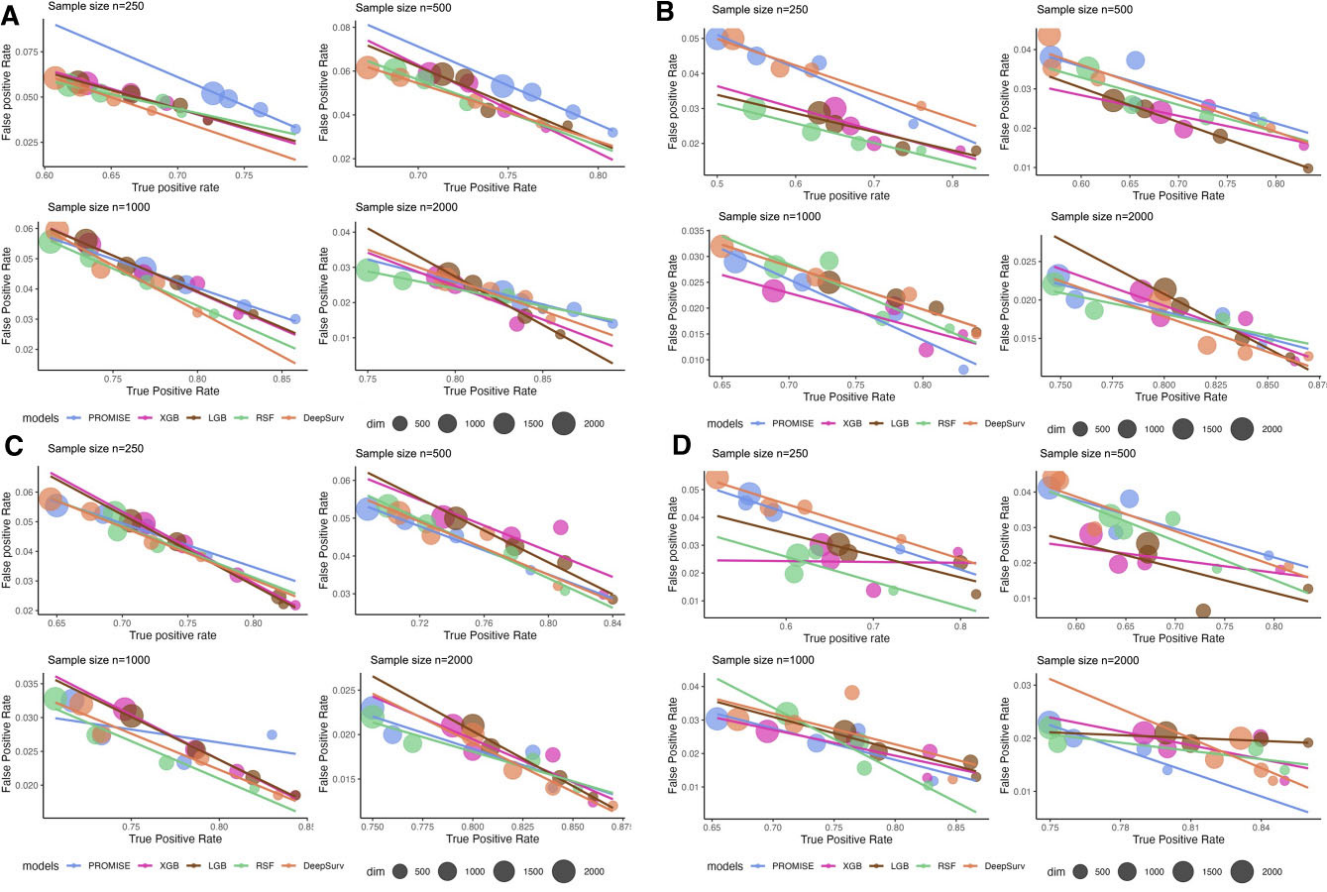
Feature selection results of different machine learning methods with different sample sizes and feature dimensions in (**A**) linear model (scenario 1), (**B**) quadratic model (scenario 2), (**C**) nonlinear model without interactions (scenario 3) and (**D**) nonlinear model with interactions (scenario 4). The size of the dot represents the corresponding feature dimension from 500 to 2000.

Overall, all approaches have yielded high TPR and low FPR in a low-dimensional setting. The results revealed a larger discrepancy as the model complexity increased from scenario 1 to scenario 4. As expected, PROMISE, which only allows linear effects, exhibited the best performance in terms of TPR and FPR in the linear setting (scenario 1) but achieved the worst performance in nonlinear scenarios when compared to other methods. The four machine learning approaches (XGB, LGB, RSF and DeepSurv) achieved comparable outcomes in scenarios 2–4, with LGB slightly outperforming the others, followed by XGB and DeepSurv. We further run the simulation experiments with four sample size settings: 250, 500, 1000 and 2000. For each sample size, we vary the feature dimension *p* and set the true signal number *q* to 2% of the feature dimension. There are thus four sets of *p* and *q*: $( {p,q} )\ \in$ {(500, 10), (1000, 20), (1500, 30), (2000, 40)}. Figure 3 shows the results from 100 replicates in each simulation scenario, with panels (A), (B), (C) and (D) depicting the results from scenarios 1, 2, 3 and 4, respectively. PROMISE-Cox and DeepSurv perform worse than the other methods in the small sample size setting (*n* = 250), while in the large sample size (*n* = 2000) setting, DeepSurv outperforms the other machine learning methods in most circumstances. However, when the sample size is between 500 and 1000, it is difficult to tell the method with the optimal TRP and FPR. The fitted line in Figure 3 illustrates the pattern of the different machine learning methods in TPR and FPR as the feature size increases. As expected, the feature selection performances of all methods decrease as the feature dimension increases, but LGB appears to be the most robust method. Similar to the low-dimensional scenario, PROMISE-Cox outperforms other methods in scenario 1, whereas the other methods exhibit a similar pattern as shown in Figure 3. Collectively, our simulations show that LGB and XGB tend to achieve superior performances when the training sample size is limited.

### Prediction performance

**Figure 4.**
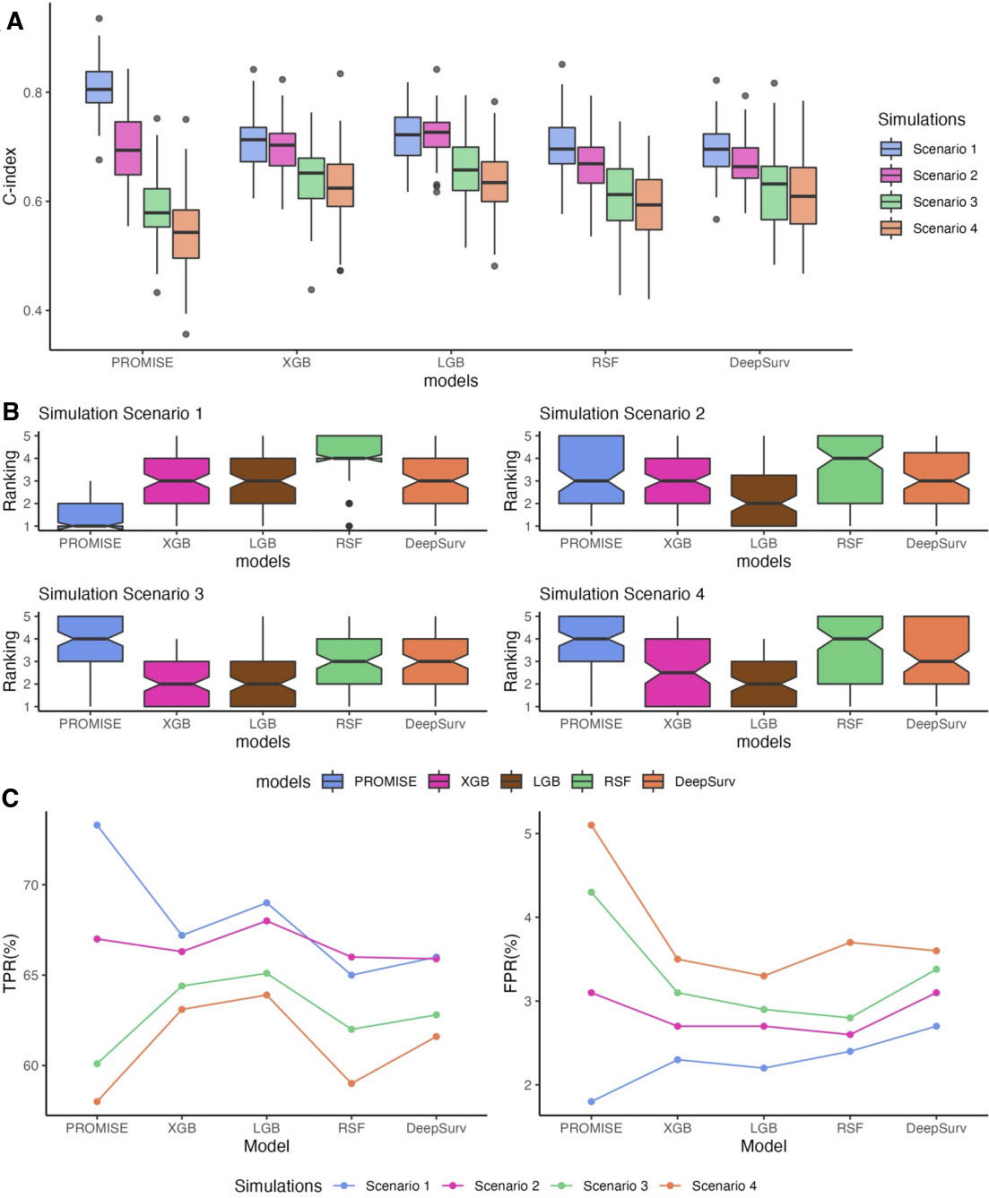
Prediction performance of different machine learning methods with sample size 500 and feature dimension 1000. (**A**) Box plot of predicted *C*-index on test data. (**B**) Ranking of the different methods based on the predicted *C*-index. (**C**) Feature selection results characterized by TPR (left) and FPR (right).

We evaluate the prediction performances of various models under four different scenarios: linear model (scenario 1), quadratic model (scenario 2), nonlinear model without interactions (scenario 3) and nonlinear model with interactions (scenario 4). In each simulation, 80% of samples were used as training data and 20% as test data. For each scenario, 100 replicates were generated. Figure 4 summarizes the results with a sample size *N* = 500, feature number *p* = 1000 and number of true signals *q* = 20. The benchmark comparison for *C*-index in the test data is reported in Figure 4A. As expected, PROMISE-Cox was consistently the top-ranked method in scenario 1 (Figure 4B and C). In all other simulation scenarios, LGB outperformed the other methods on survival prediction (Figure 4A and B) and feature selection (Figure 4C). As expected, PROMISE yielded the lowest *C*-index and the worst selection results after including more nonlinear components in scenarios 3 and 4. We also conducted simulation studies with *N* = 1000, *p* = 2000 and *q* = 40, where similar observation is noticed (Supplementary Figure S2). With the addition of more interaction terms in scenario 4, DeepSurv emerged as the overall best performing method (Supplementary Figure S1).

### Risk group classification

In this section, we further study the performances of patient risk group classification based on the following machine learning methods: XGB, LGB, RSF and DeepSurv. In simulated data, 2000 features were generated from a normal distribution with three groups of means ${\mu }_i$: ${\mu }_1 = 5,\ {\mu }_2 = 10$ and ${\mu }_3 = 15$, with each group containing 400 samples.

**Figure 5.**
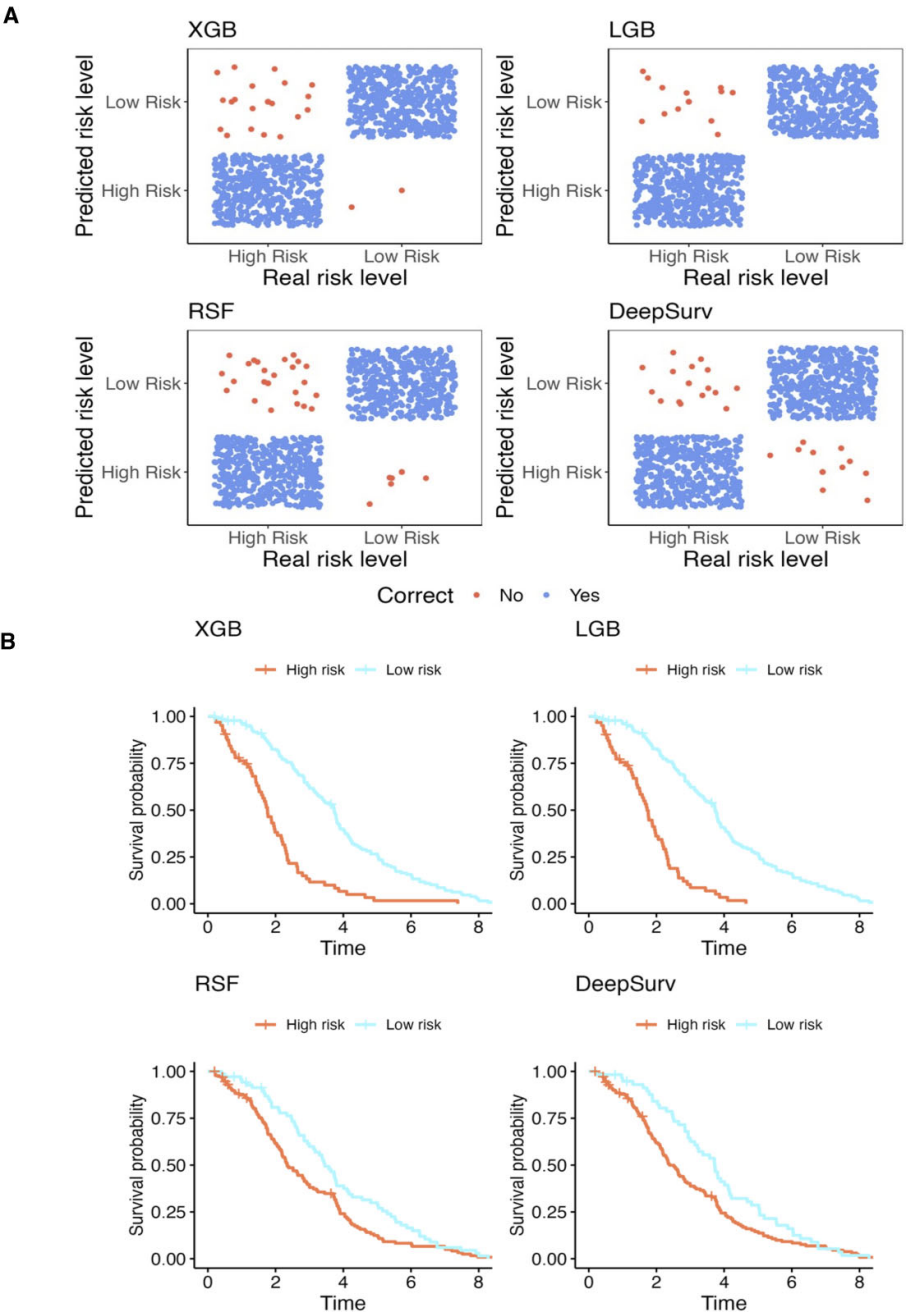
Survival calibration results for different machine learning methods. (**A**) Prediction accuracy for risk levels. (**B**) Comparison of Kaplan–Meier curves between real risk groups and predicted risk groups.

As shown in Figure 5A, among all machine learning methods, LGB yields the best misclassification rate. As expected, the predicted survival probability in the ‘low-risk’ group is significantly better than the ‘high-risk’ group in all machine learning methods (Figure 5B), with LGB demonstrating a clearer distinction between two groups.

### Identifying prognostic biomarkers in HNSCC with different modalities

**Figure 6.**
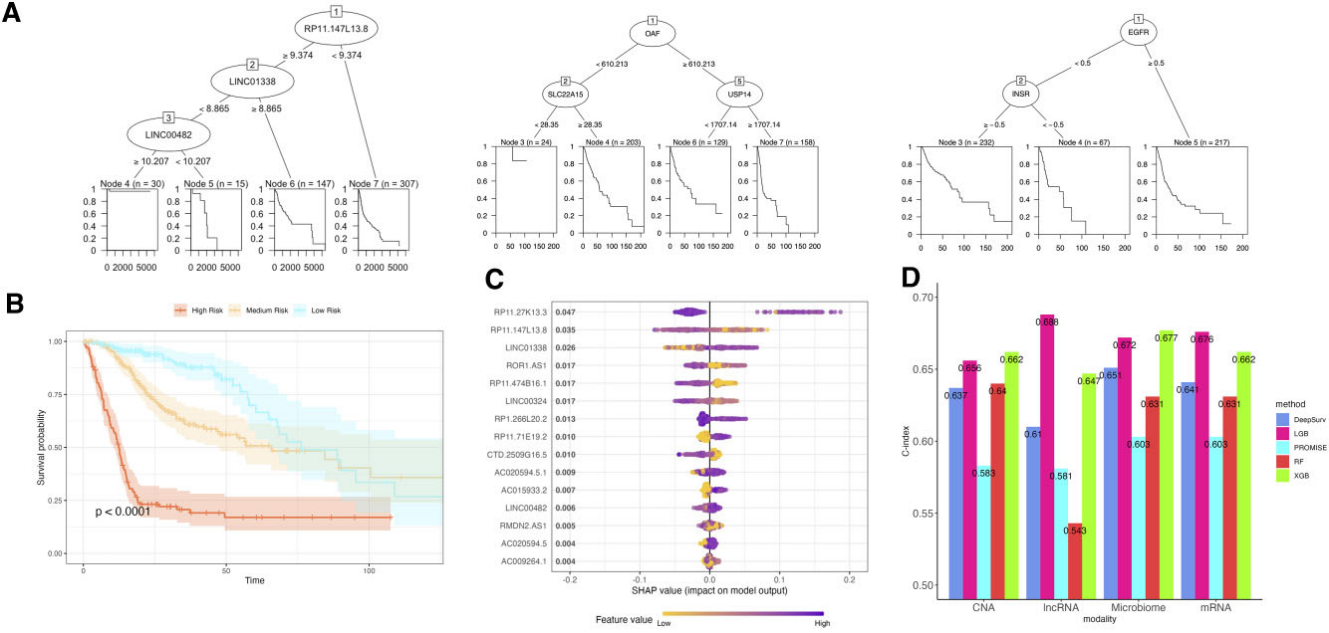
HNSCC patients’ survival prediction based on the prediction-oriented prognostic biomarker selection model. (**A**) Recursive partitioning survival trees based on the selected biomarkers in different modalities: lncRNA, mRNA and CNA. (**B**) Kaplan–Meier plots for patient subgroups stratified by risk groups characterized with the final model. (**C**) Top 15 lncRNAs calculated by SHAP values. (**D**) *C*-index of each model on validation dataset in different modalities.

Here, using The Cancer Genome Atlas head and neck squamous cell carcinoma (HNSCC) dataset, we demonstrate the use of survival machine learning methods for biomarker prioritization. Patients with three clinical covariates (age, sex and stage) and 15 878 long noncoding RNAs (lncRNAs), 1406 microbiome covariates, 20 518 mRNAs and 25 101 Copy Number alterations (CNAs) are included in the discovery datasets. The sample size for each modality is 499, 514, 515 and 516, respectively. The survival outcome had a censoring rate of around 55% for different modalities. For each different category, we randomly select 80% of samples of the original data as training data and the remaining 20% samples as the test data. When evaluating the performance of various machine learning methods, we focused on *C*-index and IBS of the test dataset. As shown in Figure 6D, LGB yielded the best *C*-index in the extremely high-dimensional cases for lncRNA, mRNA and CNA. Similar results regarding the performances of different methods on IBS are shown in Supplementary Figure S3, where LGB had the smallest values for extremely high-dimensional cases and XGB performed best on microbiome modality for a moderate dimensional case. We highlighted the overlapped top 3 markers that were selected by multiple methods in Table 3. LncRNAs have emerged as potential prognostic biomarkers for predicting therapeutic outcomes in cancer (26,27). Notably, the results from LGB-based models contain more overlapped lncRNAs than any other methods, demonstrating their high reliability on biomarker selection, while the XGB-based model outperforms the other methods with moderate feature dimension (*p* = 1406). As shown in Figure 6A, higher gene expression values of RP11.147L13.8 and LINC00482 are associated with better survival in HNSCC patients, while a higher value of LINC01338 predicts worse survival. As illustrated in Figure 6B, survival curves for patient subgroups stratified according to the LGB model’s predicted risk groups demonstrate a significant disparity in survival with *P*-value <0.0001. The SHAP value plot (Figure 6C) (28) reveals that RP11.147L13.8 and LINC01338 are also listed among top 3 prognostic biomarkers. Their prognostic values have also been reported in other cancer types (29,30). For other modalities, some significant biomarkers recognized for HNSCC in recent studies, such as EGFR (31) and USP14 (32), were identified by the LGB-based models, and both EGFR and USP14 show negative influence on patients’ survival with larger values (Figure 6A).

**Table 3.** Top 3 biomarkers detected by different machine learning methods of different modalities (markers that are selected by multiple methods are highlighted in bold)

Modality	PROMISE-Cox	XGB-Cox	LGB-Cox	RSF	DeepSurv
lncRNA	RP5.1061H20.4	**C5orf66.AS1**	**RP11.27K13.3**	RP11.664I21.6	**RP11.27K13.3**
	**LINC00324**	RP11.25I15.3	**RP11.147L13.8**	RP11.12K6.2	**LINC01281**
	RP11.114H20.1	RP11.664H17.1	**LINC01281**	**RP11.147L13.8**	RP11.70D24.2
Microbiome	***Hylemonella***	*Anaerococcus*	*Mesoplasma*	*Prevotella*	*Actibacterium*
	Polyomavirus	***Hylemonella***	*Turicella*	*Nitrosococcus*	***Anaerococcus***
	***Alphapapillomavirus***	***Alphapapillomavirus***	*Caldimonas*	*Haematobacter*	*Legionella*
mRNA	**PTX3**	RNASEN	OAF	**HDAC4**	EGFP
	PCMT1	MAML2	SLC22A15	PRIMA1	PMP22
	CBX3	**HDAC4**	USP14	GTPBP1	**PTX3**
CNA	C11orf91	PMP22	**EGFR**	**EGFR**	SIPA1L3
	MYO1G	USP14	INSR	ZNF146	ADTRP
	CSMD1	LARGE-AS1	CDKN2B-AS1	SLCO1A2	**EGFR**

## DISCUSSION

In this study, we compared the performance of the prediction-oriented prognostic biomarker selection framework based on five machine learning methods: PROMISE-Cox, XGB-Cox, LGB-Cox, RSF and DeepSurv. Our findings suggest that the prediction-based biomarker selection strategy is a viable option for biomarker discovery. Consistent with previous findings (33,34), PROMISE-Cox outperforms other approaches in linear models, while XGB-Cox and LGB-Cox perform better in the nonlinear high-dimensional scenarios. Additionally, our simulation results indicate that LGB-Cox is recommended when the sample size is <500 and the feature number is >1000, whereas DeepSurv tends to deliver better results when the sample size is further increased (e.g. when *n* > 2000). Given the fact that most cancer genomic studies have sample size <1000, we recommend use of boosting-based methods as the main framework for prognostic biomarker discovery in oncology.

It is important to acknowledge the fact that hyperparameter tuning during the model deployment can have a significant impact on the final performance. In this aspect, RSF and PROMISE-Cox methods do provide advantages in terms of the number of hyperparameters, although the computational burden in PROMISE-Cox will increase considerably when more resampling steps are incorporated into the nested CV and SS steps. While XGB-Cox and LGB-Cox have more hyperparameters than RSF and PROMISE, they are more manageable than deep learning methods in the parameter tuning burden. Based on both our experience and results from this study, we recommend no more than eight layers in DeepSurv (including four dense layers and four dropout layers) and consider less than four layers in the initial run when the sample size is between 250 and 1000. To ensure reproducibility, we have provided functions in the package Xsurv (7) that enables the automatic parameter tuning process.

Modern supervised machine learning methods are designed to deliver the predictive models efficiently by incorporating the training data as a whole. Due to their black-box nature, the performances of these new methods in reliably selecting true signal features or prioritizing candidate biomarkers, especially in the context of survival models, have been largely understudied. Although some software packages can provide metrics on feature importance (such as information gain or SHAP values in XGB/LGB, feature importance in RSF and importance scores in deep learning models), the information on the number of selected features in the final model is often not readily accessible. The proposed prediction-oriented feature selection framework combines the machine learning methods with a robust feature selection procedure based on CV and the top-*k* selection procedure. We demonstrate that this framework outperforms PROMISE in terms of feature selection and prediction results in nonlinear model settings. In comparison to conventional machine learning methods for survival data, the proposed framework provides more meaningful insight into the importance of the candidate biomarkers.

Although we focused on the biomarker identification based on the Cox models, the prediction-oriented framework combined with machine learning methods can be extended to a variety of other survival models, including the accelerated failure time (AFT) model (35), censoring unbiased deep learning (36) and alternative deep learning methods such as deep survival machines (37). The AFT model, in particular, has been implemented in the Xsurv package for implementing XGB-Cox and LGB-Cox, and is thus readily available and utilized to fulfill the proposed framework.

## DATA AVAILABILITY

HNSCC data underlying this article are available in the Cancer Genome Atlas Program(TCGA) database. Data can be found in PanCancer Atlas publication pages (https://gdc.cancer.gov/about-data/publications/pancanatlas) and can be downloaded using cBioPortal at (https://www.cbioportal.org/study/summary?id=hnsc_tcga_pan_can_atlas_2018). Code and scripts used to generate the results are available in the Zenodo doi: (https://doi.org/10.5281/zenodo.7991272).

## Supplementary Material

lqad055_Supplemental_File
