# Supplementary material for: Prediction-oriented prognostic biomarker discovery with survival machine learning methods
Source: NAR Genom Bioinform. 2023 Jun 16;5(2):lqad055. doi: 10.1093/nargab/lqad055 (PMC10273194; doi:10.1093/nargab/lqad055)
Supplement: lqad055_Supplemental_File [file lqad055_supplemental_file.pdf]

Supplementary Material for Prediction-oriented prognostic biomarker discovery with survival machine learning methods

SUPPLEMENTARY MATERIALS

PROMISE Algorithm

Let $S^{\Lambda} = \{j: \beta_j \neq 0, j = 1, \dots, p\}$ be the set of indices of non-zero coefficients that estimated under tuning parameter Λ. By sub-sampling the data for B times, we can estimate the selection probability by:

$$\widehat{\text{Pr}}_j^{\Lambda} = \frac{1}{B}\sum_{b=1}^{B} 1\{j \in S_b^{\Lambda}\},$$

where S_b^{Λ} is the selected features in the *b*th sub-sample using Λ. Following the same setting in [Soyeon,2018], we set the sub-sample size equal to $[\frac{n}{2}]$. With a cutoff $0 < p_{cut} < 1$ and parameter set $\Delta = \{\Lambda_r = (\lambda_r, \alpha_r), r = 1, \dots, R\}$, the estimated SS set is given by,

$$\hat{S}_{ss} = \left\{j: \max_{\Lambda \in \Delta} \widehat{\text{Pr}}_j^{\Lambda} > p_{cut}\right\}.$$

PROMISE leverage the advantages from both CV and SS strategies by coupling them in one unified procedure. The sequence of PROMISE steps in the context of Cox model are summarized below:

Supplementary Table 1

Algorithm 1: PROMISE
1.Set tuning parameter set $\boldsymbol{\Delta} = \{\boldsymbol{\Lambda_r} = (\boldsymbol{\lambda_r}, \boldsymbol{\alpha_r}), \boldsymbol{r} = \mathbf{1}, \dots, \boldsymbol{R}\}$ and cutoff set $\boldsymbol{\Gamma} = \{\boldsymbol{p_{cut,m}}, \boldsymbol{m} = \mathbf{1}, \dots, \boldsymbol{M}\}$
2.Perform K-fold CV:
(a) Split data into K parts.
(b) For each part, using the samples except the kth fold where k from 1 to K.
(i) Sub-sampling B times with sample size equal to half of the sample. Fit the model with all $\boldsymbol{\Lambda} \in \boldsymbol{\Delta}$. Calculate $\widehat{\text{Pr}}_{\boldsymbol{j}}^{\boldsymbol{\Lambda}}$ for each $\boldsymbol{\Lambda}$.
(ii) Using the combination of $\boldsymbol{p_{cut,m}}$ and $\boldsymbol{\Lambda_r}$ to select the features and then estimate the model correspondingly.
(c) Get C-index by predicting the model in (ii) on the kth fold data.
(d) Find the best combination of $\boldsymbol{p_{cut,m}}$ and $\boldsymbol{\Lambda_r}$ with highest C-index
3. Obtain the final model.
(a) Perform B times sub-sampling in entire train data.
(b) Use the best $\boldsymbol{p_{cut,m}}$ and $\boldsymbol{\Lambda_r}$ to select the final features.
(c) Obtain the final model by using the selected features to fit.
end

Prediction-oriented feature selection framework with top-k

Let $\Omega = \{1, \ldots, \omega\}$ where $\omega \leq N \wedge p$ and $F = \{1, \ldots, f\}$ where π is the total number of filtered models. By given the number $\tau \in \Omega$, we can calculate the frequency of feature j's ranking not exceeding τ. We denote the frequency as f_j^τ. Hence, we can define the feature selection set S by setting a threshold value $\pi \in F$,

$$S_\pi^\tau = \{j: f_j^\tau \geq \pi\}.$$

The sequence of machine learning based prediction-oriented feature selection framework with top-k can be summarized below:

Supplementary Table 2

Algorithm 2: prediction-oriented feature selection framework with top-k
1.Perform K-fold CV:
(a) Split data into K parts.
(b) For each part, using the samples except the kth fold where k from 1 to K:
(i) Sub-sampling B times with sample size equal to half of the sample. Default value for B is 100.
(ii) Fit the machine learning models in subsamples with random parameters.
(c) Get C-index by predicting the models (with different random hyper-parameters) in (ii) on the kth fold data and filter the models with C-index larger than c, where c is the threshold, and the default value of c is 0.6.
(d) Set the searching set for $\boldsymbol{\tau}$ and $\boldsymbol{\pi}$. The default set for $\boldsymbol{\tau}$ is {10,20,…,50} and $\boldsymbol{\pi}$ is {0.5,0.6,…,1}.
(e) Get feature selection sets $\boldsymbol{S_\pi^\tau}$ with different combination of $\boldsymbol{\tau}$ and $\boldsymbol{\pi}$.
2. Feature selection:
(a) Refit the models with different $\boldsymbol{S_\pi^\tau}$ in 5-fold CV. The fold number can be changed.
(b) Get $\boldsymbol{S_{best}}$ with highest C-index value.
3. Obtain the final model:
(a) Fit the final model by using the features in $\boldsymbol{S_{best}}$ on entire train data.
end

Supplementary Figures

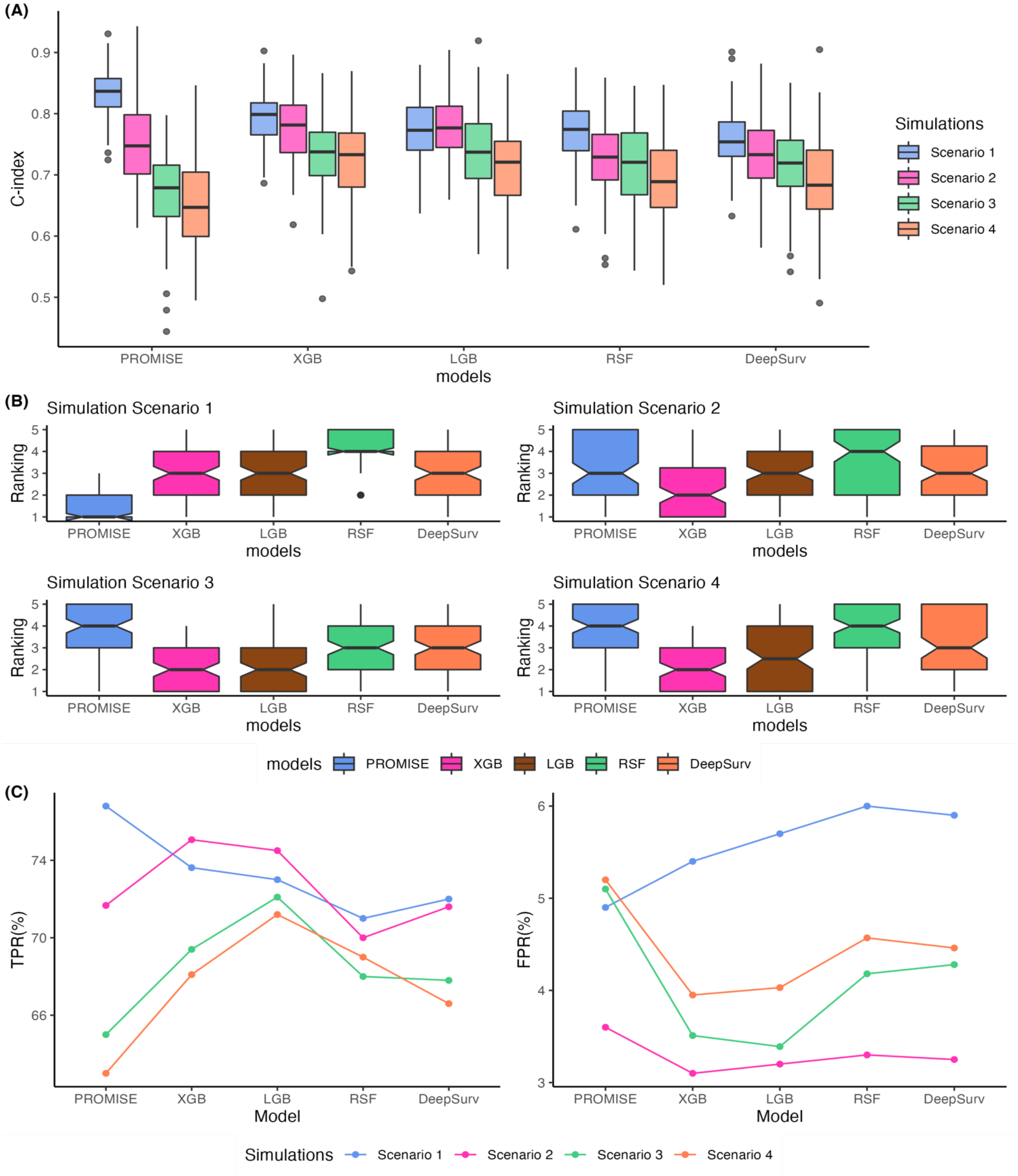

Supplementary Figure 1. Prediction performance of different machine learning methods with sample size of 1000 and feature dimension of 2000. (A) Comparison of C-index among different models and simulation scenarios. (B) Ranking of the different methods according to their predicted C-index. (C) Comparison of TPR (left) and FPR (right) from feature selection results.

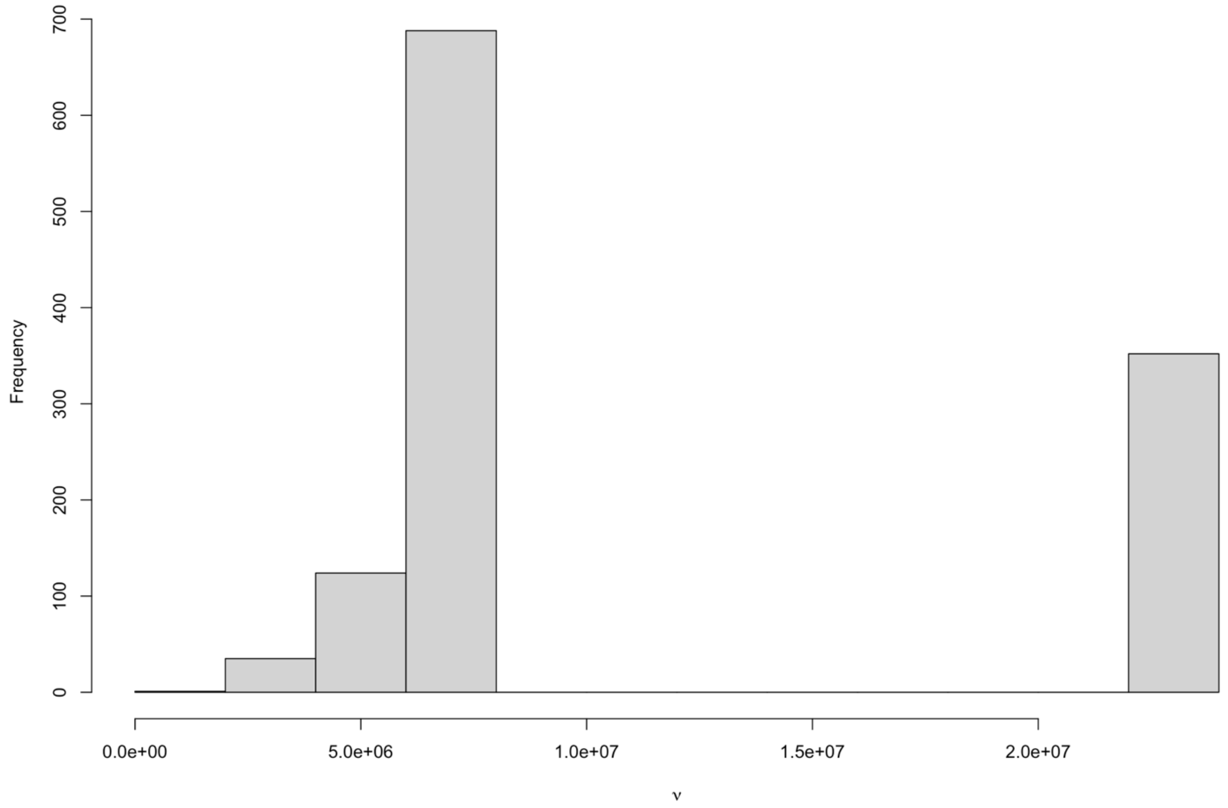

Supplementary Figure 2. The distribution of ν for risk group classification simulation corresponding to three different risk groups.

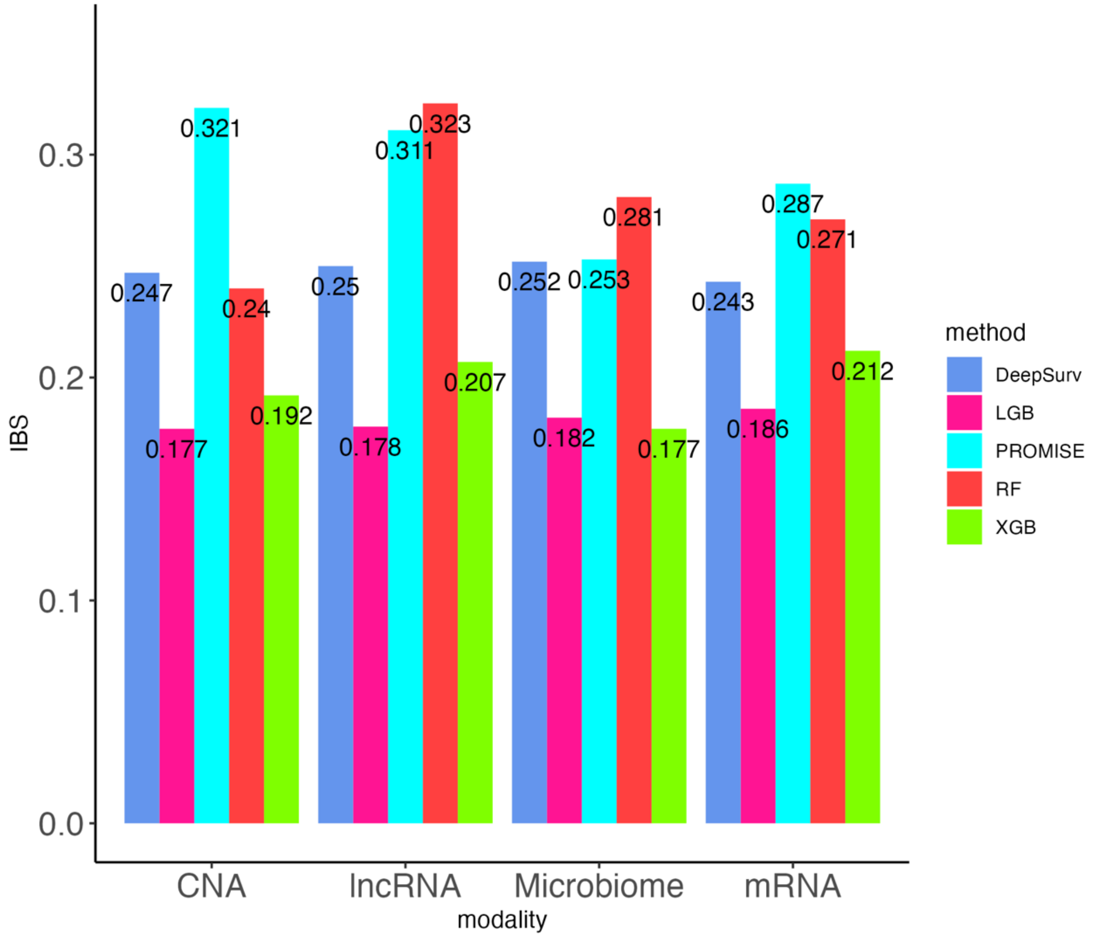

Supplementary Figure 3. IBS of each model on validation dataset in different modalities.
